# Analysis of trends in sepsis mortality in Brazil and by regions from 2010 to 2019

**DOI:** 10.11606/s1518-8787.2022056003789

**Published:** 2022-04-11

**Authors:** Nyara Rodrigues Conde de Almeida, Giovana Fonseca Pontes, Felipe Lima Jacob, João Victor Salvador Deprá, João Pedro Pires Porto, Fernanda Rocha de Lima, Mário Roberto Tavares Cardoso de Albuquerque

**Affiliations:** I Universidade Federal do Pará Faculdade de Medicina Belém PA Brasil Universidade Federal do Pará. Faculdade de Medicina. Belém, PA, Brasil; II Centro Universitário do Estado do Pará Faculdade de Medicina Belém PA Brasil Centro Universitário do Estado do Pará. Faculdade de Medicina. Belém, PA, Brasil; III Universidade do Estado do Pará Faculdade de Medicina Belém PA Brasil Universidade do Estado do Pará. Faculdade de Medicina. Belém, PA, Brasil; IV Centro Universitário do Estado do Pará Belém PA Brasil Centro Universitário do Estado do Pará. Módulo de Interação em Saúde na Comunidade. Belém, PA, Brasil

**Keywords:** Sepsis, epidemiology, Hospital Mortality, trends, Risk Factors, Socioeconomic Factors, Unified Health System

## Abstract

**OBJECTIVE:**

To characterize the profile of inpatients and trend of sepsis mortality in the Brazilian Unified Health System (SUS), throughout Brazil, and in its regions separately, from 2010 to 2019.

**METHODS:**

Observational, analytical and retrospective study of secondary data obtained through consultation to the *Sistema de Informação Hospitalar* (Hospital Information System). All incoming septicemia notifications from January 1, 2010 to December 31, 2019 were included. The following sociodemographic variables were used: sex, age, race, region and federative unit of residence. For data analysis, we used mortality and hospitalization coefficient, relative risk and Joinpoint regression.

**RESULTS:**

There were a total of 1,044,227 cases of sepsis in Brazil, yielding a mean prevalence coefficient of 51.3/100 thousand inhabitants. There were 463,000 deaths from sepsis recorded, with a mean prevalence coefficient of 22.8 deaths/100,000 inhabitants. The highest rates occurred among the elderly, of brown race, and there was no significant difference between genders. The Southeast region accounted for the highest rates of hospitalization and deaths. A general trend toward increased mortality was observed in the period studied.

**CONCLUSION:**

The heterogeneity of Brazil should be considered regarding socioeconomic and demographic characteristics, and differences in health investment and underreporting between regions, in order to understand the disease’s epidemiological course. Finally, these findings should be correlated with other studies, in an effort to understand the behavior of the disease, and provide inputs for public and private policies in order to reduce the expressiveness of cases and deaths from sepsis in Brazil.

## INTRODUCTION

Sepsis is an inflammatory response syndrome, caused by an infection that may originate in one place and cause systemic changes in an attempt to fight it, requiring prompt recognition and early treatment^1,2^.

According to the Spread study, one-third of intensive care unit (ICU) beds are occupied by patients with severe sepsis and septic shock, with an overall lethality of 55%^3^. Similarly, sepsis contributes between one-third and one-half of deaths in the United States hospitals. This figure reflects and justifies these admissions surpassing admissions for myocardial infarction and stroke^4^. In Brazil, the prevalence of sepsis reaches 30%, and an in-hospital mortality rate is close to 55%, being characterized as the main cause of death in non-cardiac ICUs^2,5^.

It is a complex condition that requires a variety of equipment, medications, and a specialized team, being the main cost generator in both public and private systems^1^. In the United States, a patient with sepsis spends about US$38,000, while in Brazil the mean daily hospital expense is US$1,028, depending on the severity and length of stay^6^. Therefore, we can observe the impact of the disease, mainly on the public health system, considering the massive occupation of beds, and the costly treatment.

The underlying cause of death is defined as injury or disease that triggered a succession of factors that culminated in death, being relevant in public health for planning actions of prevention and promotion^7^. When designated as sepsis, this cause loses specificities of the origin and characterization of diagnosis, recording little useful information, being defined as garbage code - intermediate or final causes that do not identify relevant characteristics about the first diagnosis^8,9^.

The Society of Critical Care Medicine, the European Society of Intensive Care Medicine, the American College of Chest Physicians, the American Thoracic Society and the Surgical Infection Society held a consensus conference that resulted in the adoption of a sepsis stratification model, known as “PIRO”, which refers to the following factors: P - predisposition, I - infection, R - response and O - organ dysfunction^10^. In this sense, predisposition to sepsis is related to patients with advanced age and/or comorbidities; infection is caused mostly by bacteremia; response to infection is characterized as hypoxemia or septic shock; and organ dysfunction is exemplified as dysfunction of the lungs with acute respiratory syndrome, and kidneys with acute renal failure^11^.

In this context, the actions aimed to reduce the number of severe cases and to provide access to the health system are extremely relevant, as well as the training of the medical team working at emergency services. All that allows, in addition to early identification and treatment of patients, an updated search for recommended therapies and protocols.

Thus, it is perceived a need for further studies on the epidemiological behavior and analysis of the trend of hospitalizations and deaths from sepsis in Brazil, in order to overcome the lack of this type of research, and thus reduce the shortage of updated information on the subject. It is expected to contribute with results that may ensure better characterization of the disease, and serve as a study basis for future productions, diagnosis, treatment and prognosis of the disease.

This study thus aims to characterize the profile of hospitalized patients and the trend of mortality from sepsis in the Brazilian Unified Health System (SUS) in Brazil and its regions, from 2010 to 2019.

## METHODS

This is an observational, analytical, retrospective study of secondary data, obtained through consultation to the databases of health information in the *Sistema de Informação Hospitalar* (Hospital Information System - SIH/SUS).

We included all the SIH notifications for hospitalizations and deaths, with diagnosis referring to the PIRO code in the international statistical ranking of diseases and health-related issues, ICD 10, sepsis (A40-A41), and with hospitalization dated between January 1, 2010 and December 31, 2019.

To describe the epidemiological profile of patients and hospitalizations, the following variables were selected: sex, age, race, region, and federative unit of residence.

To quantify the population, we considered the 2010 demographic census and data from the *Pesquisa Nacional por Amostra de Domicílios* (National Household Sample Survey - PNAD) for intercensal years, provided by the *Instituto Brasileiro de Geografia e Estatística* (Brazilian Institute of Geography and Statistics - IBGE).

For data analysis, the mortality coefficient and hospitalization coefficient, expressed per 100 thousand inhabitants, were standardized by the direct standardization method in order to reduce the influence of external variations over the time period evaluated. For that, population was standardized to a reference year chosen in the middle of the time interval studied (2014). Rates were adjusted using population as denominator in the respective strata of sex, age group, race/color, Region and Federation Unit. The annual mean mortality and hospitalization coefficient was calculated with the numerator as the mean number (mean from 2010 to 2019) of deaths and hospitalizations, respectively, applied to the reference population (2014), and the denominator was the inhabitants of the respective stratum in the reference year.

To compare the risk of death between different strata of the population, the relative risk (RR) was used, and its confidence interval (95%CI) was used as the effect size estimate. The expression of statistical results with effect size and CI provides a more comprehensive method to interpret these results, not only in terms of statistical significance, but also the extent of the effects^12^.

To assess the time trend of the standardized mortality coefficients, and to check the significance of the most recent changes in the patterns of these coefficients, we used Poisson Joinpoints regression (inflection point analysis). This is a non-linear regression method that adjusts a series of trend lines over the analyzed period, and provides the calculation of the annual percentage change (APC) for each trend in the period, and the average annual percentage change (AAPC) for the entire period^13^. The APC characterizes the rate under analysis over time, assuming that the rate in a year is a constant percentage of the rate in the previous year, while the AAPC is a summary-measure for the entire period, a weighted average of all the APCs in the regression model.

The GraphPad Prism Version 6.01 was used to calculate the relative risk, its confidence interval, and the confidence intervals of the standardized coefficients. For the Joinpoint regression analysis, the Joinpoint Regression Program, version 4.9.0.0, was used.

Since this is a study in which information was collected in secondary databases and of public domain, the research did not have to be submitted to and approved by a Research Ethics Committee, respecting the norms for research involving human beings (Res. CNS 466/12) of the *Conselho Nacional de Saúde* (Brazilian National Health Council, CNS).

## RESULTS

From 2010 to 2019, 463,000 deaths from sepsis were recorded in Brazil (Table 1). The standardized mean coefficient of death from sepsis was 22.8 per 100,000 inhabitants (95%CI: 22.6–23.0). Of the total, 51.4% of deaths were of men, and 48.6% were women. It is noteworthy that the risk of death in females compared to males was close to one, suggesting that probabilities of death in both sexes were similar. Regarding age group, the highest death rate was found in the elderly (≥ 60 years), with 112.9 deaths per 100,000 inhabitants, followed by the age group 50 to 59 years, rate of 24 deaths/100,000 (95CI: 23.4–24.6), and under four years of age, 13 deaths/100,000 (95%CI: 12.4–13.7). The probability of death was 5.6 times higher among the elderly compared to the five to nine years age group. The North was the region with lowest death rate, with a mortality coefficient equal to 12.1 deaths/100,000 inhabitants, while the highest rates occurred in the Southeast (30.6 deaths/100,000 inhabitants), and the South (25.8 deaths/100,000 inhabitants).

**Table 1:** Deaths and standardized mortality ratios (per 100,000 inhabitants), stratified by sex, age group, race/color and Great Region, Brazil, 2010–2019.

Variable	Deaths		Mortality coeff.^a^		Relative Risk (RR)	
		
	n	%	Coeff.	95%CI	RR	95%CI
Total deaths	462,971	100	22.8	22.6–23.0	-	-
Sex						
Male	237,891	51.4	24.1	23.8–24.4	Ref.	-
Female	225,080	48.6	21.5	21.2–21.7	1.04	1.04–1.05
Age range (years)						
≤ 4	17,291	3.7	13.0	12.4–13.7	1.06	1.01–1.11
5 to 9	1,724	0.4	1.2	1.0–1.4	Ref.	-
10 to 19	5,113	1.1	1.5	1.4–1.7	1.57	1.49–1.65
20 to 29	9,272	2.0	2.9	2.7–3.1	2.24	2.13–2.35
30 to 39	16,047	3.5	5.0	4.8–5.3	3.01	2.88–3.16
40 to 49	30,006	6.5	10.8	10.5–11.2	3.76	3.59–3.94
50 to 59	55,628	12.0	24.0	23.4–24.6	4.35	4.15–4.55
≥ 60	327,890	70.8	114.1	112.9–115.4	5.65	5.40–5.91
Race^b^						
White	176,097	54.6	19.3	19.0–19.6	1.09	1.09–1.10
Black	18,804	5.8	10.7	10.2–11.2	1.17	1.15–1.18
Brown	127,450	39.5	13.7	13.4–13.9	Ref.	-
Great Region						
North	20,962	4.5	12.1	11.6–12.6	Ref.	-
Northeast	84,701	18.3	15.1	14.8–15.4	1.10	1.09–1.11
Southeast	262,337	56.7	30.6	30.3–31.0	1.30	1.29–1.31
South	75,262	16.3	25.8	25.3–26.4	1.02	1.01–1.03
Midwest	19,709	4.3	12.8	12.2–13.4	1.14	1.13–1.16

Coeff.: coefficient; 95%CI: confidence interval of 95%.

^a^ Mean annual coefficient obtained by the direct standardization method, where numerator was the mean number (mean from 2010 to 2019) of deaths applied to the reference population (2014), and the denominator was the population of the respective stratum in this reference year.

^b^ Information not available for all races. Population information for sex, age group, race/color, and Great Region were obtained from the IBGE 2010 Demographic Census or from estimates from the PNAD for intercensal years.

Comparing the regions with their respective federative units (Table 2), in the Southeast the highest death rate was in São Paulo, with 34.2 per 100,000 inhabitants. In the South, the highest rate occurred in Rio Grande do Sul (30.8 deaths/100,000 inhabitants). In the Northern region, the highest rate was in Amazonas (16.2 deaths/100,000 inhabitants). In Northeast, the highest rate occurred in Pernambuco (27.8 deaths/100,000 inhabitants). Finally, in the Midwest the highest rate of deaths was found in Mato Grosso (22.6 deaths/100,000 inhabitants).

**Table 2:** Deaths and standardized mortality ratios (per 100,000 inhabitants), stratified by Federative Unit, Brazil, 2010–2019.

Variable	Deaths		Mortality coeff.^a^		Relative Risk (RR)	
		
	n	%	Coeff.	95%CI	n	%
Total deaths	462,971	100	22.8	22.6–23.0	-	-
North Region						
Rondônia	2,397	0.5	13.9	12.2–15.7	1.07	1.02–1.12
Acre	400	0.1	5.0	3.6–6.9	1.30	1.20–1.42
Amazonas	6,265	1.4	16.2	15.0–17.5	1.77	1.71–1.83
Roraima	298	0.1	5.8	3.9–8.3	1.36	1.24–1.50
Pará	9,703	2.1	11.9	11.1–12.7	1.25	1.21–1.29
Amapá	295	0.1	3.8	2.6–5.5	1.43	1.30–1.57
Tocantins	1,604	0.3	10.6	9.0–12.4	1.70	1.62–1.78
Northeast Region						
Maranhão	6,025	1.3	8.7	8.1–9.5	1.52	1.47–1.57
Piauí	2,275	0.5	7.1	6.2–8.1	1.61	1.55–1.68
Ceará	17,483	3.8	19.6	18.7–20.5	1.91	1.85–1.97
Rio Grande do Norte	5,423	1.2	15.9	14.6–17.3	1.25	1.20–1.29
Paraíba	6,002	1.3	15.3	14.1–16.5	1.00	0.97–1.04
Pernambuco	25,956	5.6	27.8	26.7–28.9	1.70	1.65–1.75
Alagoas	3,638	0.8	11.0	9.9–12.2	Ref.	-
Sergipe	2,460	0.5	11.0	9.6–12.4	1.95	1.88–2.03
Bahia	15,439	3.3	10.4	9.9–10.9	1.40	1.36–1.44
Southeast Region						
Minas Gerais	58,459	12.6	28.2	27.5–28.9	1.27	1.24–1.31
Espírito Santo	5,342	1.2	13.8	12.6–15.0	1.09	1.06–1.13
Rio de Janeiro	47,165	10.2	28.2	27.4–29.0	2.04	1.98–2.10
São Paulo	151,371	32.7	34.2	33.6–34.7	2.08	2.02–2.14
South Region						
Paraná	27,516	5.9	24.8	23.8–25.7	1.47	1.42–1.51
Santa Catarina	13,221	2.9	19.4	18.4–20.5	1.31	1.27–1.35
Rio Grande do Sul	34,525	7.5	30.8	29.8–31.9	1.39	1.35–1.43
Midwest Region						
Mato Grosso do Sul	2,393	0.5	9.1	8.0–10.4	1.63	1.57–1.70
Mato Grosso	7,425	1.6	22.6	21.0–24.3	1.65	1.60–1.71
Goiás	5,067	1.1	7.6	7.0–8.3	1.36	1.32–1.41
Distrito Federal	4,824	1.0	16.9	15.4–18.4	1.69	1.64–1.75

Coeff.: coefficient; 95%CI: confidence interval of 95%.

^a^ Mean annual coefficient obtained by the direct standardization method, where numerator was the mean number (mean from 2010 to 2019) of deaths applied to the reference population (2014), and the denominator was the population of the respective stratum in this reference year. Population information per federative unit were obtained from the IBGE 2010 Demographic Census or from estimates from the PNAD for intercensal years.

Regarding hospitalizations, in the study period there were a total of 1,044,227 cases of sepsis in Brazil (Table 3), with mean prevalence coefficient of 51.3 per 100,000 inhabitants (95%CI: 51.0–51.6), being slightly higher in males, 55.5% of cases. The elderly group (60 years or older) had the highest rate of hospitalizations, 192.1 cases per 100,000 inhabitants (95%CI: 190.5–193.7), followed by children up to four years old, 117.4 per 100,000 inhabitants (95%CI: 115.6–119.3). Regarding race, whites accounted for 388,330 notifications for sepsis, followed by browns, with 306,687 hospitalizations. Regarding this variable, it is important to consider that approximately 28% of the data were classified as missing information for race, both for the number of hospitalizations and total deaths. The highest percentage of hospitalizations occurred in the Southeast, 51.6% of the cases, and the highest rates of hospitalizations in the South and Southeast, both above 60 cases per 100,000 inhabitants.

**Table 3:** Hospitalizations and standardized hospitalization coefficients (per 100,000 inhabitants), stratified by sex, age group, race/color and Great Region, Brazil, 2010–2019.

Variable	Hospitalizations		Hospitalization coeff.^a^	
	
	n	%	Coeff.	95%CI
Total hospitalizations	1,044,227	100	51.3	51.0–51.6
Sex				
Male	547,269	52.4	55.5	55.0–56.0
Female	496,958	47.6	47.4	47.0–47.8
Age range (years)				
≤ 4	155,541	14.9	117.4	115.6–119.3
5 to 9	16,422	1.6	11.4	10.8–11.9
10 to 19	31,094	3	9.4	9.1–9.8
20 to 29	39,464	3.8	12.5	12.1–12.9
30 to 39	50,744	4.9	15.9	15.5–16.3
40 to 49	75,970	7.3	27.4	26.8–28.1
50 to 59	121,939	11.7	52.5	51.5–53.4
≥ 60	553,053	53	192.1	190.5–193.7
Race^b^				
White	388,330	52.9	42.5	42.1–42.9
Black	38,764	5.3	22.2	21.5–22.9
Brown	306,687	41.8	32.9	32.6–33.3
Greats Region				
North	56,001	5.4	32.4	31.5–33.2
Northeast	205,837	19.7	36.8	36.3–37.3
Southeast	539,158	51.6	62.9	62.4–63.5
South	197,240	18.9	67.8	66.8–68.7
Midwest	45,991	4.4	29.9	29.0–30.8

Coeff.: coefficient; 95%CI: confidence interval of 95%.

^a^ Mean annual coefficient obtained by the direct standardization method, where numerator was the mean number (mean from 2010 to 2019) of hospitalizations applied to the reference population (2014), and the denominator was the population of the respective stratum in this reference year.

^b^ Information not available for all races. Population information for sex, age group, race/color, and Great Region were obtained from the IBGE 2010 Demographic Census or from estimates from the PNAD for intercensal years.

As for the states, in the Southeast, Minas Gerais had the highest rate of hospitalizations, 81.3 cases of sepsis per 100,000 inhabitants (95%CI: 80.0–82.5). In the other regions, the highest rates of hospitalizations also corresponded to the same states with highest rates of deaths (Table 4).

**Table 4:** Hospitalizations and standardized hospitalization coefficients (per 100,000 inhabitants), stratified by Federative Unit, Brazil, 2010–2019.

Variable	Hospitalizations		Hospitalization coeff.^a^	
	
	n	%	Coeff.	IC95%
Total hospitalizations	1,044,227	100	51.3	51.0–51.6
North Region				
Rondônia	8,229	0.8	47.5	44.3–50.9
Acre	1,128	0.1	14.1	11.6–17.0
Amazonas	13,011	1.2	33.8	32.0–35.7
Roraima	804	0.1	16	12.7–19.9
Pará	28,602	2.7	35.1	33.8–36.4
Amapá	759	0.1	10.1	7.9–12.6
Tocantins	3,468	0.3	23.1	20.7–25.6
Northeast Region				
Maranhão	14,572	1.4	21.2	20.1–22.3
Piauí	5,185	0.5	16.1	14.7–17.6
Ceará	33,625	3.2	37.7	36.5–39.0
Rio Grande do Norte	15,973	1.5	46.9	44.7–49.3
Paraíba	21,962	2.1	56.2	53.8–58.5
Pernambuco	56,022	5.4	60.1	58.5–61.7
Alagoas	13,359	1.3	40.5	38.4–42.7
Sergipe	4,633	0.4	20.6	18.8–22.6
Bahia	40,506	3.9	27.3	26.5–28.1
Southeast Region				
Minas Gerais	168,546	16.1	81.3	80.0–82.5
Espírito Santo	17,937	1.7	46.3	44.2–48.5
Rio de Janeiro	84,965	8.1	50.9	49.8–52.0
São Paulo	267,710	25.6	60.4	59.7–61.1
South Region				
Paraná	68,893	6.6	62	60.5–63.5
Santa Catarina	37,116	3.6	54.6	52.8–56.4
Rio Grande do Sul	91,231	8.7	81.5	79.8–83.2
Midwest Region				
Mato Grosso do Sul	5,377	0.5	20.5	18.9–22.4
Mato Grosso	16,506	1.6	50.5	48.1–53.0
Goiás	13,647	1.3	20.5	19.4–21.7
Distrito Federal	10,461	1	36.6	34.4–38.9

Coeff.: coefficient; 95%CI: confidence interval of 95%.

^a^ Mean annual coefficient obtained by the direct standardization method, where numerator was the mean number (mean from 2010 to 2019) of hospitalizations applied to the reference population (2014), and the denominator was the population of the respective stratum in this reference year. Population information per federative unit were obtained from the IBGE 2010 Demographic Census or from estimates from the PNAD for intercensal years.

In the trend analysis of mortality rate (Table 5), a general upward trend was observed throughout the studied period (2010–2019, AAPC 7.4, 95%CI: 6.1–8.7). Two main trends were found in the country: a sharply increasing trend in the period from 2010 to 2016 (AAPC 9.1, 95%CI: 7.5–10.8), followed by a trend also increasing, but less sharply, in the period from 2016 to 2019 (AAPC 4.0, 95%CI: 0.2–8.0). According to the population strata, it was observed that temporal pattern in both male and female groups followed the national pattern, with a period of sharp growth in the years 2010–2016 (APC close to the country’s APC of 9.1), and a period of less sharp growth in the years 2016–2019 (APC of 4.0 equal to the country’s APC). As for age groups, the elderly showed the sharpest APC in the period 2010–2016 (APC 8.4, 95%CI: 7.2–9.6), while in the period 2010–2014 the age group five to nine years showed a significant reduction in mortality (APC -6.8, 95%CI -9.4 to -4.2). As for the regions, the Northeast stood out experiencing the highest increase in mortality among all regions in the period 2010–2016, with APC 17.8 (95%CI: 14.5–21.2). No region disclosed significant trend of mortality decline.

**Table 5:** Joinpoint regression analysis of standardized sepsis mortality coefficients stratified by sex, age group, race, and Region, Brazil, 2010–2019.

Variable	Trend 1			Trend 2			Total period	
		
	Period	APC	95%CI	Period	APC	95%CI	AAPC	95%CI
Brazil – Total	2010–2016	9.1^a^	7.5 to 10.8	2016–2019	4.0^a^	0.2 to 8.0	7.4^a^	6.1 to 8.7
Sex								
Female	2010–2016	9.2^a^	7.3 to 11.2	2016–2019	4.0	-0.7 to 8.8	7.4^a^	5.9 to 9.0
Male	2010–2016	9.1^a^	7.8 to 10.4	2016–2019	4.0^a^	0.8 to 7.2	7.3^a^	6.3 to 8.4
Age range (years)								
≤ 4	2010–2015	-2.6^a^	-4.3 to -0.8	2015–2019	-0.1	-2.9 to 2.8	-1.5^a^	-2.7 to -0.3
5 to 9	2010–2014	-6.8^a^	-9.4 to -4.2	2014–2019	3.1^a^	1.1 to 5.2	-1.4^a^	-2.7 to -0.2
10 to 19	2010–2016	3.0^a^	2.2 to 3.7	2016–2019	-2.3	-5.1 to 0.6	1.2^a^	0.4 to 2.0
20 to 29	2010–2015	7.2^a^	4.1 to 10.4	2015–2019	-0.3	-4.1 to 3.7	3.8^a^	2.0 to 5.7
30 to 39	2010–2015	5.8^a^	2.7 to 9.0	2015–2019	-0.8	-4.5 to 3.2	2.8^a^	1.0 to 4.7
40 to 49	2010–2016	4.8^a^	4.1 to 5.4	2016–2019	0.3	-1.6 to 2.2	3.3^a^	2.7 to 3.8
50 to 59	2010–2016	5.0^a^	3.6 to 6.4	2016–2019	0.4	-3.7 to 4.7	3.4^a^	2.2 to 4.7
≥ 60	2010–2016	8.4^a^	7.2 to 9.6	2016–2019	1.9	-1.4 to 5.2	6.2^a^	5.1 to 7.2
Race								
White	2010–2019	7.9^a^	7.1 to 8.8				7.9^a^	7.1 to 8.8
Brown	2010–2019	12.0^a^	10.1 to 14.0				12.0^a^	10.1 to 14.0
Black	2010–2013	2.4	-16.9 to 26.3	2013–2019	12.8^a^	8.2 to 17.6	9.2^a^	3.1 to 15.7
Great Region								
North	2010–2019	7.7^a^	5.8 to 9.7				7.7^a^	5.8 to 9.7
Northeast	2010–2016	17.8^a^	14.5 to 21.2	2016–2019	1.1	-6.0 to 8.6	11.9^a^	9.4 to 14.6
Southeast	2010–2015	8.1^a^	5.8 to 10.4	2015–2019	4.6^a^	2.0 to 7.3	6.5^a^	5.2 to 7.9
South	2010–2019	6.3^a^	5.8 to 6.9				6.3^a^	5.8 to 6.9
Midwest	2010–2016	8.2^a^	6.1 to 10.3	2016–2019	4.2	-1.2 to 9.9	6.9^a^	5.1 to 8.7

APC: average percentage change AAPC: annual average percentage change 95%CI: confidence interval of 95%.

^a^ Significantly different from zero (p < 0.005).

## DISCUSSION

Sepsis is a serious health issue in Brazil and worldwide, being a challenge to be faced by public policies. Recent studies have shown a tendency for this syndrome to increase in the national scenario, highlighting several factors that contribute to this context. Among these, we highlight the increase in the Brazilian population concomitantly with the increase in life expectancy, exposing a larger number of patients with chronic and immunosuppressed diseases^14,15^.

As for mortality, 462,971 deaths from the disease were reported in the same period, with a mortality coefficient of 22.8 deaths per 100,000 inhabitants (95%CI: 22.6–23.0). Several reasons may be associated with the high mortality rate from sepsis in Brazil, including the possible neglect of sepsis by healthcare professionals, thus hindering and delaying the disease treatment, leading to increased mortality. Moreover, another associated factor would be the absence of intermediate care units in the Brazilian hospitals, which would lead to longer ICU stays for patients, and increased exposure to and prevalence of sepsis^3^.

Compared to other countries, both developed and developing ones, deaths from sepsis in Brazil are in a global trend of high prevalence. A survey conducted in Spain showed a trend of increasing cases of sepsis in the country, and a prevalence of hospitalizations of 57 cases per 100,000 inhabitants. This figure is similar to the findings of this study about the Brazilian reality (51.3 cases per 100,000 inhabitants). This fact shows that, although developed countries have better financial conditions to afford public health costs, sepsis is a serious problem to be faced worldwide^16^.

The Brazilian Spread research, a multicenter study conducted by ILAS, evaluated the prevalence and mortality from severe sepsis and septic shock in 2015, when the increase in mortality from sepsis in Brazil was most evident^3^. Moreover, reviewing a study that observed the rates of deaths from sepsis by regime in the regions of Brazil from 2011 to 2017, these higher rates were observed in all regions except the South. Most deaths occur in the public health sector, accounting for about 60% of deaths. This evidences the need for improvements in public health^17^. Moreover, the high prevalence in some states may be related to the high number of inhabitants in the region^18^, such as São Paulo, the most populous state in Brazil, where 25.6% of the sepsis cases occurred.

In this study, 51 (23%) of the 227 hospitals reviewed had low availability of resource. It explains how short investment in the means necessary for diagnosis and treatment of sepsis directly interferes with mortality from the disease. This fact is exemplified by the Southeast, which received an investment of R$216,979,860.32, and the Midwest, with R$15,798,077.40^18^.

Moreover, the Brazilian regions with lowest hospitalization rates were the Midwest (4.4%), and North (5.4%). Similarly, these regions accounted for the lowest mortality rates, totaling 12.1 deaths/100,000 inhabitants in the North (95%CI: 11.6–12.6) and 12.8 deaths/100,000 inhabitants in the Midwest (95%CI: 12.2–13.4). Given this, one possible reason for the lower number of hospitalizations and deaths is the lower population density of these regions, and the lower number of elderly (over 60 years) and, consequently, carriers of chronic diseases that favor the development of sepsis. It is also important to highlight the hypothesis of underreporting of hospitalizations and deaths from sepsis, causing omission of important epidemiological data, since such underreporting may be associated with lack of medical care, resources for diagnosis of underlying cause, and incorrect reports^19^.

Despite having the lowest rates of hospitalizations and deaths in the country, the Midwest Region, according to the Spread study, has the highest mortality rate with 70%, while the Southeast has the lowest, 51.2%. In this scenario, the relationship between high mortality rates from sepsis in Brazil and low levels of investment in ICU was demonstrated in an epidemiological study published by The Lancet, which showed the association between low investment and high mortality rates^3^.

It is also worth noting the figures for sepsis in the Northeast and South regions. The Northeast, the second region with the highest number of hospitalizations, totaled 19.7% of the national contingent, with a coefficient of 36.8 hospitalizations per 100,000 inhabitants (95%CI: 36.3–37.3), followed by the South totaling 18.9%, with hospitalization coefficient equal to 67.8 per 100,000 inhabitants (95%CI: 66.8–68.7). In this context, the Northeast region was responsible for the second highest number of deaths from the disease, 18.3% of the national total, followed by the South region, with 16.3% of the deaths.

Among the states in the Northeast, the highest percentage of hospitalization was identified in Pernambuco (5.4%), with a mortality rate of 27.8 per 100,000 inhabitants (95%CI: 26.7–28.9). This fact corroborates an epidemiological study 2015 as reference year, in which the state was also found to be the most affected one, with 7,861 hospitalizations and 44.94% mortality rate^18^. Moreover, a remarkable percentage difference between the states of Pernambuco and Piauí may be observed when looking at hospitalization rates, in which Pernambuco accounts for 5.4% of notifications, and Piauí, 0.5%. This disparity may be related to the population difference between both states, with the state of Pernambuco’s population approximately twice as large^20^.

In Brazil, no statistically relevant variation was observed in the number of hospitalizations for men and women, accounting for 52.4% of cases in males, against 47.6% in females. The numbers referring to deaths followed the same pattern, with males representing 51.4%, and females 48.6%. Considering that the relative risk of death among females was 1.05, it cannot be said that there is a predominance of deaths from either sex. A systematic review covering a decade of literature states that studies pointing to gender-related mortality and sepsis are inconclusive^21^. In reviewing literature, some findings pointed to male prevalence, including a retrospective study of quantitative approach, conducted in Brazil, in which male prevalence was perceived and documented in a sample with 347 patients^22^. In this sense, this study is considered to corroborate statistical accuracy in the Brazilian scientific scenario.

The temporal pattern of mortality rate trends for both sexes showed sharp growth throughout the analyzed time period, showing alignment with the national pattern (2010–2019, AAPC 7.4, 95%CI: 6.1–8.7).

These data diverge from a study reviewing the worldwide incidence of sepsis between 1990 and 2017, which found a regression in the number of deaths during the period. It showed that in the period studied, the incidence of sepsis decreased 18.8%, and mortality from sepsis decreased 29.7%. In the same article, there is a large variation in mortality rates from sepsis among different countries (Latin America and Africa report the highest rates), which is explained by the inefficiency of health systems in sites with more deaths in preventing, identifying, and treating sepsis. However, further studies are needed to better understand this disparity^23^.

Moreover, in countries with high incidence of sepsis such as Brazil, many cases are suspected to be due to nosocomial infection (patients acquiring infections in hospitals), due to invasive procedures or inadequate hand washing of health care workers. It is thus assumed that Brazil is still in negative projections regarding global parameters, needing measures for identification, control and prevention of sepsis.

When analyzing the age range of individuals who died from sepsis, the highest rate corresponds to the group over 60 years (70.8%), with a mortality rate of 114.1 deaths per 100,000 inhabitants, and a relative risk of 5.65 (95%CI: 5.40–5.91), followed by the group from 50 to 59 years (12%), with 24 deaths per 100,000 inhabitants. Accordingly, in a study analyzing 848 patients admitted to the ICU with severe sepsis and septic shock, elderly patients accounted for 62.6% of the inpatients, and with the highest APACHE III score (Acute Physiology and Chronic Health Evaluation), in addition to longer hospital stay, which is explained by the greater presence of chronic diseases, comorbidities, frailty, and functional impairment in these individuals^24^.

Moreover, the elderly group showed a general trend of increased mortality between 2010 and 2019 (AAPC 6.2, 95%CI: 5.1–7.2). In this context, a review of literature on the epidemiology of sepsis in the United States found that more than half the cases of severe sepsis occur in individuals over 65 years of age, because this population is the most affected by chronic diseases, increasing the risk of mortality from sepsis^25^.

It can also be said that aging, by decreasing the production of cytokines and causing changes in adaptive immunity, makes individuals more vulnerable to septic conditions. Moreover, post-menopausal women, due to the loss of estrogen, present structural changes in the genitourinary system, being more susceptible to develop urinary tract infection, which, according to the Spread study, represents one of the four main causes of infection that evolves to sepsis, followed by pulmonary and intra-abdominal infection^26,3^. Therefore, the trend of increasing mortality among the elderly is evident in the national scenario, considering the aging population and the exposure to risk factors^14^.

In the age group under four years, a considerable number of hospitalizations is observed, 14.9% of the total, second only to the over-60s (53%), and an important, but less expressive, value in the number of deaths, with 3.7% of the total, behind the age groups over 40 years. Similarly, a meta-analysis, conducted in 2018, in which the global impact of pediatric and neonatal sepsis between 1979 and 2016 was evaluated, postulated that mortality in children and neonates ranged between 1% and 5% in cases of sepsis, and between 9% and 20% in severe sepsis^27^. Moreover, an online seminar involving experts in pediatric internal medicine in 2017 indicated that in the United States the incidence of neonatal sepsis is one to four per thousand live births, in addition to relating the lower birth weight with higher probability of developing a septic condition (10.96 cases per thousand live births for newborns weighing between 401 and 1,500g)^28^. Thus, one can see the significant number of hospitalizations for neonatal and pediatric sepsis in Brazil, although mortality is within the global range, requiring attention from health institutions for these parameters.

When analyzing the racial variable, considering the white, black and brown races, in relation to the mortality coefficient, the highest rate occurred in the white race, followed by the brown and black races. In this same scenario, a survey conducted in 60 municipalities in the five Brazilian regions in 2017, based on the *Sistema de Informação sobre Mortalidade* (Mortality Information System), revealed an incidence of 54.6% of total deaths from sepsis in white individuals, followed by brown (33.7%) and black (8.2%)^9^.

However, an integrative literature review on the access of the black population to health services found that this racial group suffers greater restrictions on access to health services due to several factors such as discrimination, violence, structural barriers, socioeconomic factors, work of professionals, disrespect for cultural, ethnic, and racial diversity. Consequently, much data about this share of the population is not included in the database, which can make the reading and interpretation of data referring to this group partially misleading^29^.

Moreover, according to the study *Infection Rate and Acute Organ Dysfunction Risk as Explanations for Racial Differences in Severe Sepsis*, mortality among black patients hospitalized for infection and severe sepsis was higher than in whites, explained by higher probability of hospitalization with infection and higher risk of developing acute organ dysfunction^30^. Such a discrepancy in data comparing the U.S. and Brazil may be justified by the large miscegenation of the Brazilian population, in addition to the large number of notifications missing information on race/color, and the lower access of blacks to health care.

Thus, it is important to take into account that Brazil is an extremely heterogeneous country, so the data on hospitalizations for sepsis tend to vary according to regional characteristics, given the number of inhabitants, amount of investment proposed in each federative unit and/or socioeconomic differences in each region. This study found divergences in relation to other studies and the disparities found in a country like Brazil, which may occur if the database does not contain all the updated information.

Finally, this study aimed to expand knowledge about the profile of the septic patient, and the development of the disease throughout the country. It was found that further studies are needed in order to correlate these findings, and generate greater contribution and basis for public and private policies for prevention of sepsis, early care, and resulting reduction of mortality, especially for the groups most affected by the disease, ensuring quality of life for the population.
