# Supplementary material for: Analysis of trends in sepsis mortality in Brazil and by regions from 2010 to 2019
Source: Rev Saude Publica. 2022 Apr 11;56:25. doi: 10.11606/s1518-8787.2022056003789 (PMC9004703; doi:10.11606/s1518-8787.2022056003789)
Supplement: Translation

# Translation: Análise de tendência de mortalidade por sepse no Brasil e por regiões de 2010 a 2019

**Authors:** Nyara Rodrigues Conde de Almeida, Giovana Fonseca Pontes, Felipe Lima Jacob, João Victor Salvador Deprá, João Pedro Pires Porto, Fernanda Rocha de Lima, Mário Roberto Tavares Cardoso de Albuquerque

**Affiliations:** I Brasil Universidade Federal do Pará. Faculdade de Medicina. Belém, PA, Brasil; II Brasil Centro Universitário do Estado do Pará. Faculdade de Medicina. Belém, PA, Brasil; III Brasil Universidade do Estado do Pará. Faculdade de Medicina. Belém, PA, Brasil; IV Brasil Centro Universitário do Estado do Pará. Módulo de Interação em Saúde na Comunidade. Belém, PA, Brasil

**Keywords:** Sepse, epidemiologia, Mortalidade Hospitalar, tendências, Fatores de Risco, Fatores Socioeconômicos, Sistema Único de Saúde

## Abstract

**OBJETIVO:**

Caracterizar o perfil dos pacientes internados e a tendência de mortalidade por sepse no Sistema Único de Saúde (SUS), em todo Brasil e em suas regiões separadamente, entre os anos de 2010 e 2019.

**MÉTODOS:**

Estudo observacional, analítico e retrospectivo de dados secundários obtidos por consulta ao Sistema de Informação Hospitalar. Foram incluídas todas as notificações por septicemia admitidas entre 1 de janeiro de 2010 e 31 de dezembro de 2019. Utilizou-se as variáveis sociodemográficas: sexo, idade, raça, região e unidade federativa de residência. Para a análise dos dados, utilizou-se coeficiente de mortalidade e de internação, risco relativo e regressão por *Joinpoints*.

**RESULTADOS:**

Totalizaram-se 1.044.227 casos de sepse no país, perfazendo um coeficiente de prevalência média de 51,3/100 mil habitantes. Foram registrados 463 mil óbitos por sepse, com coeficiente médio de 22,8 óbitos/100 mil habitantes. As maiores taxas ocorreram entre os idosos, de raça parda e não houve uma diferença significativa entre os sexos. A Região Sudeste foi responsável pelo maior índice de internação e óbitos. Observou-se uma tendência geral de aumento da mortalidade no período estudado.

**CONCLUSÃO:**

Cabe considerar a heterogeneidade do Brasil, no que concerne às características socioeconômicas e demográficas e às diferenças de investimento em saúde e de subnotificações entre as regiões, a fim de entender o traçado epidemiológico da doença. Por fim, é necessário correlacionar esses achados com demais estudos, buscando entendimento do comportamento da doença e embasamento para políticas públicas e privadas, com intuito de diminuir a expressividade de casos e óbitos por sepse no país.

## INTRODUÇÃO

A sepse é uma síndrome de resposta inflamatória, causada por uma infecção que pode se originar em um local e causar alterações sistêmicas na tentativa de combatê-la, exigindo um pronto reconhecimento e tratamento precoce^1,2^.

De acordo com o estudo *Spread*, um terço dos leitos das unidades de terapia intensiva (UTI) são ocupados por pacientes com sepse grave e choque séptico, apresentando letalidade global de 55%^3^. Do mesmo modo, a sepse contribui entre um terço e metade dos óbitos nos hospitais dos Estados Unidos, dado que reflete e justifica que essas internações superaram as admissões por infarto do miocárdio e acidente vascular cerebral^4^. Em todo o Brasil, a prevalência de sepse chega a 30% e uma taxa de mortalidade hospitalar próxima de 55%, caracterizando-se como principal causa de morte nas UTIs não cardiológicas^2,5^.

Trata-se de uma condição complexa que necessita de diversos equipamentos, medicamentos e uma equipe especializada, sendo a principal geradora de custos no sistema público e particular^1^. Nos Estados Unidos, um paciente com sepse despende cerca de US$ 38 mil e, no Brasil, o gasto hospitalar médio diário é de US$ 1.028, variando de acordo com a gravidade e tempo de internação^6^. Logo, pode-se observar o impacto da doença, principalmente, ao sistema público de saúde, considerando a grande ocupação de leitos e o tratamento custoso.

A causa básica da morte define-se como lesão ou doença que iniciou uma sucessão de fatores que culminaram no óbito, apresentando relevância na saúde pública para planejamentos de ações de prevenção e promoção^7^. Essa causa, quando assinada como sepse, perde especificidades da origem e caracterização do diagnóstico, registrando-se uma informação pouco útil, definindo-se como código *garbage* – causas intermediárias ou finais que não identificam características relevantes sobre o primeiro diagnóstico^8,9^.

A *Society of Critical Care Medicine*, a *European Society of Intensive Care Medicine*, o *American College of Chest Physicians*, a *American Thoracic Society* e a *Surgical Infection Society* realizaram uma conferência de consenso, da qual o resultado foi a adoção de um modelo de estratificação da sepse, conhecido como “PIRO”, que faz referência aos seguintes fatores: P – predisposição, I – infecção, R – resposta e O – disfunção do orgão^10^. Nesse sentido, a predisposição à sepse relaciona-se a pacientes com idade avançada e/ou comorbidades; a infecção é causada, na maior parte, por bacteremia; a resposta à infecção se caracteriza como hipoxemia ou choque séptico; e a disfunção do órgão exemplifica-se como disfunção dos pulmões, com síndrome respiratória aguda e dos rins, com insuficiência renal aguda^11^.

Nesse contexto, as ações voltadas para redução do número de casos graves e para acesso ao sistema de saúde são de extrema relevância, bem como a capacitação da equipe médica que atua nos serviços de emergência. Tudo isso permite, além de identificar e tratar precocemente os pacientes, a busca atualizada por terapias e protocolos recomendados.

Assim, entende-se que há a necessidade de mais estudos sobre o comportamento epidemiológico e a análise da tendência das internações e dos óbitos por sepse no Brasil, com o intuito de suprir a carência desse tipo de pesquisa e, portanto, reduzir o déficit de informação atualizada sobre a temática, esperando contribuir com resultados que possam garantir uma melhor caracterização da doença e servir como base de estudo para produções futuras, diagnóstico, tratamento e prognóstico da doença.

Assim, este estudo visa caracterizar o perfil dos pacientes internados e a tendência de mortalidade por sepse no Sistema Único de Saúde (SUS) no Brasil e suas regiões, entre os anos de 2010 e 2019.

## MÉTODOS

Trata-se de um estudo observacional, analítico e retrospectivo de dados secundários, obtidos por meio de consulta às bases de dados das informações de saúde no sistema de informação hospitalar (SIH/SUS).

Foram incluídas todas as notificações, para internações e óbitos, do SIH cujo diagnóstico referia-se ao código PIRO na classificação estatística internacional de doenças e problemas relacionados à saúde, CID 10, septicemia (A40-A41) e com data de internação dentro do período compreendido entre 1 de janeiro de 2010 a 31 de dezembro de 2019.

Para descrição do perfil epidemiológico dos pacientes e das internações, foram selecionadas as seguintes variáveis: sexo, idade, raça, região e unidade federativa de residência.

Para quantificação da população, considerou-se o censo demográfico de 2010 e os dados da Pesquisa Nacional por Amostra de Domicílios (PNAD) para os anos intercensitários, fornecidos pelo Instituto Brasileiro de Geografia e Estatística (IBGE).

Para a análise dos dados, o coeficiente de mortalidade e o coeficiente de internação, expressos por 100 mil habitantes, foram padronizados pelo método direto de padronização, a fim de diminuir a influência de variações externas no decorrer do tempo avaliado. Para isso, padronizou-se a população para um ano de referência escolhido no centro do intervalo de tempo estudado (2014). As taxas foram ajustadas tendo como denominador a população nos respectivos estratos de sexo, faixa etária, raça/cor, Região e Unidade da Federação. Então, o coeficiente de mortalidade e de internação anual médio foi calculado com o numerador como número médio (média de 2010 a 2019) de mortes e internações, respectivamente, aplicadas à população de referência (2014) e o denominador foi a população do respectivo estrato no ano de referência.

Para comparar o risco de óbito entre diferentes estratos da população, foi utilizado o risco relativo (RR) e seu intervalo de confiança (IC95%) foi usado como estimativa do tamanho do efeito. A expressão de resultados estatísticos com tamanho de efeito e IC fornece um método interpretativo mais abrangente desses resultados, não apenas em termos de significância estatística, como também a extensão dos efeitos^12^.

Para avaliar a tendência temporal dos coeficientes padronizados de mortalidade e verificar a significância das mudanças mais recentes nos padrões desses coeficientes, utilizou-se a regressão por *Joinpoints* de Poisson (análise por pontos de inflexão), um método de regressão não linear que ajusta uma série de linhas de tendência no decorrer do período analisado e provê o cálculo da variação percentual anual (*annual percentual change* – APC) para cada tendência no período e a variação percentual média anual (*average annual percentual change* – AAPC) para o período inteiro^13^. O APC caracteriza a taxa em análise no decorrer do tempo, assumindo que a taxa em um ano é uma percentagem constante da taxa no ano anterior, enquanto a AAPC é uma medida-resumo do período inteiro, uma média ponderada de todas as APC do modelo de regressão.

Para o cálculo do risco relativo, seu intervalo de confiança e dos intervalos de confiança dos coeficientes padronizados foi utilizado o GraphPad Prism Versão 6.01. Para a análise de regressão por *Joinpoints*, foi utilizado o *Joinpoint Regression Program*, versão 4.9.0.0.

Por se tratar de um estudo no qual as informações foram coletadas em bases de dados secundários e de domínio público, não se fez necessária a submissão e a aprovação da pesquisa por um Comitê de Ética em Pesquisa, respeitadas as normas de pesquisa envolvendo seres humanos (Res. CNS 466/12) do Conselho Nacional de Saúde.

## RESULTADOS

No período de 2010 a 2019, foram registrados 463 mil óbitos por sepse no Brasil (Tabela 1). O coeficiente médio padronizado de morte por sepse foi de 22,8 a cada 100 mil habitantes (IC95% 22,6–23,0). Do total, 51,4% dos óbitos eram do sexo masculino e 48,6% do sexo feminino. Destaca-se que o risco de morte no sexo feminino em relação ao masculino foi próximo a um, indicando serem semelhantes as probabilidades de óbito nos dois sexos. Em relação à faixa etária, a maior taxa de óbitos foi encontrada nos idosos (≥ 60 anos), com 112,9 óbitos por 100 mil habitantes, seguidos da faixa etária de 50 a 59 anos, taxa de 24 óbitos/100 mil (IC95% 23,4–24,6) e menores de quatro anos de idade, 13 óbitos/100 mil (IC95% 12,4–13,7). A probabilidade de morte foi 5,6 vezes maior entre os idosos comparados à faixa etária de cinco a nove anos de idade. A região com menor taxa de mortes foi a Norte, com coeficiente de mortalidade igual a 12,1 óbitos/100 mil habitantes, e as maiores ocorreram no Sudeste (30,6 óbitos/100 mil hab.) e Sul (25,8 óbitos/100 mil hab.).

**Tabela 1:** Óbitos e coeficientes padronizados de mortalidade (por 100 mil habitantes), estratificados por sexo, faixa etária, raça/cor e Grande Região, Brasil, 2010–2019.

Variável	Óbitos		Coef. de mortalidade^a^		Risco relativo (RR)	
		
	n	%	Coef.	IC95%	RR	IC95%
Óbitos totais	462.971	100	22,8	22,6–23,0	-	-
Sexo						
Masculino	237.891	51,4	24,1	23,8–24,4	Ref.	-
Feminino	225.080	48,6	21,5	21,2–21,7	1,04	1,04–1,05
Grupo etário (anos)						
≤ 4	17.291	3,7	13,0	12,4–13,7	1,06	1,01–1,11
5 a 9	1.724	0,4	1,2	1,0–1,4	Ref.	-
10 a 19	5.113	1,1	1,5	1,4–1,7	1,57	1,49–1,65
20 a 29	9.272	2,0	2,9	2,7–3,1	2,24	2,13–2,35
30 a 39	16.047	3,5	5,0	4,8–5,3	3,01	2,88–3,16
40 a 49	30.006	6,5	10,8	10,5–11,2	3,76	3,59–3,94
50 a 59	55.628	12,0	24,0	23,4–24,6	4,35	4,15–4,55
≥ 60	327.890	70,8	114,1	112,9–115,4	5,65	5,40–5,91
Raça^b^						
Branca	176.097	54,6	19,3	19,0–19,6	1,09	1,09–1,10
Preta	18.804	5,8	10,7	10,2–11,2	1,17	1,15–1,18
Parda	127.450	39,5	13,7	13,4–13,9	Ref.	-
Grande Região						
Norte	20.962	4,5	12,1	11,6–12,6	Ref.	-
Nordeste	84.701	18,3	15,1	14,8–15,4	1,10	1,09–1,11
Sudeste	262.337	56,7	30,6	30,3–31,0	1,30	1,29–1,31
Sul	75.262	16,3	25,8	25,3–26,4	1,02	1,01–1,03
Centro-Oeste	19.709	4,3	12,8	12,2–13,4	1,14	1,13–1,16

Coef.: coeficiente; IC95%: intervalo de confiança de 95%.

^a^ Coeficiente anual médio obtido pelo método direto de padronização, onde o numerador foi o número médio (média de 2010 a 2019) de mortes aplicadas à população de referência (2014) e o denominador foi a população do respectivo estrato, neste ano de referência.

^b^ Informação não disponível para todas as raças. As informações populacionais de sexo, faixa etária, raça/cor e Grande Região foram obtidas do Censo Demográfico 2010 do IBGE ou das estimativas da Pesquisa Nacional por Amostra de Domicílios (PNAD) para os anos intercensitários.

Comparando as regiões com suas respectivas unidades federativas (Tabela 2), na região Sudeste, a maior taxa de óbitos ocorreu em São Paulo, perfazendo 34,2 a cada 100 mil habitantes. No Sul, a maior taxa ocorreu no Rio Grande do Sul (30,8 óbitos/100 mil habitantes). Na região Norte, a maior taxa ocorreu no Amazonas (16,2 óbitos/100 mil habitantes). No Nordeste, a maior taxa ocorreu em Pernambuco (27,8 óbitos/100 mil habitantes). Por fim, no Centro-Oeste, identificou-se a maior taxa de óbitos no Mato Grosso (22,6 óbitos/100 mil habitantes).

**Tabela 2:** Óbitos e coeficientes padronizados de mortalidade (por 100 mil habitantes), estratificados por Unidade Federativa, Brasil, 2010–2019.

Variável	Óbitos		Coef. de mortalidade^a^		Risco relativo (RR)	
		
	n	%	Coef.	IC95%	RR	IC95%
Óbitos totais	462.971	100	22,8	22,6–23,0	-	-
Região Norte						
Rondônia	2.397	0,5	13,9	12,2–15,7	1,07	1,02–1,12
Acre	400	0,1	5,0	3,6–6,9	1,30	1,20–1,42
Amazonas	6.265	1,4	16,2	15,0–17,5	1,77	1,71–1,83
Roraima	298	0,1	5,8	3,9–8,3	1,36	1,24–1,50
Pará	9.703	2,1	11,9	11,1–12,7	1,25	1,21–1,29
Amapá	295	0,1	3,8	2,6–5,5	1,43	1,30–1,57
Tocantins	1.604	0,3	10,6	9,0–12,4	1,70	1,62–1,78
Região Nordeste						
Maranhão	6.025	1,3	8,7	8,1–9,5	1,52	1,47–1,57
Piauí	2.275	0,5	7,1	6,2–8,1	1,61	1,55–1,68
Ceará	17.483	3,8	19,6	18,7–20,5	1,91	1,85–1,97
Rio Grande do Norte	5.423	1,2	15,9	14,6–17,3	1,25	1,20–1,29
Paraíba	6.002	1,3	15,3	14,1–16,5	1,00	0,97–1,04
Pernambuco	25.956	5,6	27,8	26,7–28,9	1,70	1,65–1,75
Alagoas	3.638	0,8	11,0	9,9–12,2	Ref.	-
Sergipe	2.460	0,5	11,0	9,6–12,4	1,95	1,88–2,03
Bahia	15.439	3,3	10,4	9,9–10,9	1,40	1,36–1,44
Região Sudeste						
Minas Gerais	58.459	12,6	28,2	27,5–28,9	1,27	1,24–1,31
Espírito Santo	5.342	1,2	13,8	12,6–15,0	1,09	1,06–1,13
Rio de Janeiro	47.165	10,2	28,2	27,4–29,0	2,04	1,98–2,10
São Paulo	151.371	32,7	34,2	33,6–34,7	2,08	2,02–2,14
Região Sul						
Paraná	27.516	5,9	24,8	23,8–25,7	1,47	1,42–1,51
Santa Catarina	13.221	2,9	19,4	18,4–20,5	1,31	1,27–1,35
Rio Grande do Sul	34.525	7,5	30,8	29,8–31,9	1,39	1,35–1,43
Região Centro-Oeste						
Mato Grosso do Sul	2.393	0,5	9,1	8,0–10,4	1,63	1,57–1,70
Mato Grosso	7.425	1,6	22,6	21,0–24,3	1,65	1,60–1,71
Goiás	5.067	1,1	7,6	7,0–8,3	1,36	1,32–1,41
Distrito Federal	4.824	1,0	16,9	15,4–18,4	1,69	1,64–1,75

Coef.: coeficiente; IC95%: intervalo de confiança de 95%.

^a^ Coeficiente anual médio obtido pelo método direto de padronização, onde o numerador foi o número médio (média de 2010 a 2019) de mortes aplicadas à população de referência (2014) e o denominador foi a população do respectivo estrato, neste ano de referência. As informações populacionais por unidade federativa foram obtidas do Censo Demográfico 2010 do IBGE ou das estimativas da Pesquisa Nacional por Amostra de Domicílios (PNAD) para os anos intercensitários.

Em relação às internações, nesse período de estudo totalizaram-se 1.044.227 casos de sepse no país (Tabela 3), perfazendo um coeficiente de prevalência média de 51,3 por 100 mil habitantes (IC95% 51,0–51,6), sendo um pouco maior no sexo masculino, 55,5% dos casos. O grupo dos idosos (60 anos ou mais) foi o que sofreu o maior coeficiente de internações, 192,1 casos por 100 mil habitantes (IC95% 190,5–193,7), seguido das crianças até quatro anos de idade, 117,4 por 100 mil habitantes (IC95% 115,6–119,3). No que se refere às raças, a cor branca somou 388.330 notificações por sepse, seguida da cor parda, 306.687 internações. Sobre essa variável, é importante considerar que aproximadamente 28% dos dados foram classificados como sem informação para raça, tanto para o montante de internações como para o total de óbitos. O maior percentual de internações ocorreu na região Sudeste, 51,6% dos casos, e os maiores coeficientes de internações nas regiões Sul e Sudeste, ambas acima de 60 casos por 100 mil habitantes.

**Tabela 3:** Internações e coeficientes padronizados de internação (por 100 mil habitantes), estratificados por sexo, faixa etária, raça/cor e Grande Região, Brasil, 2010–2019.

Variável	Internações		Coef. de internação^a^	
	
	n	%	Coef.	IC95%
Internações totais	1.044.227	100	51,3	51,0–51,6
Sexo				
Masculino	547.269	52,4	55,5	55,0–56,0
Feminino	496.958	47,6	47,4	47,0–47,8
Grupo etário (anos)				
≤ 4	155.541	14,9	117,4	115,6–119,3
5 a 9	16.422	1,6	11,4	10,8–11,9
10 a 19	31.094	3	9,4	9,1–9,8
20 a 29	39.464	3,8	12,5	12,1–12,9
30 a 39	50.744	4,9	15,9	15,5–16,3
40 a 49	75.970	7,3	27,4	26,8–28,1
50 a 59	121.939	11,7	52,5	51,5–53,4
≥ 60	553.053	53	192,1	190,5–193,7
Raça^b^				
Branca	388.330	52,9	42,5	42,1–42,9
Preta	38.764	5,3	22,2	21,5–22,9
Parda	306.687	41,8	32,9	32,6–33,3
Grande Região				
Norte	56.001	5,4	32,4	31,5–33,2
Nordeste	205.837	19,7	36,8	36,3–37,3
Sudeste	539.158	51,6	62,9	62,4–63,5
Sul	197.240	18,9	67,8	66,8–68,7
Centro-Oeste	45.991	4,4	29,9	29,0–30,8

Coef.: coeficiente; IC95%: intervalo de confiança de 95%.

^a^ Coeficiente anual médio obtido pelo método direto de padronização, onde o numerador foi o número médio (média de 2010 a 2019) de internações aplicadas à população de referência (2014) e o denominador foi a população do respectivo estrato, neste ano de referência.

^b^ Informação não disponível para todas as raças. As informações populacionais de sexo, faixa etária, raça/cor e Grande Região foram obtidas do Censo Demográfico 2010 do IBGE ou das estimativas da Pesquisa Nacional por Amostra de Domicílios (PNAD) para os anos intercensitários.

Quanto aos estados, na região Sudeste, Minas Gerais perfez o maior coeficiente de internações, 81,3 casos de sepse por 100 mil habitantes (IC95% 80,0–82,5). Nas demais regiões, as maiores taxas de internações também corresponderam aos mesmos estados das maiores taxas de óbitos (Tabela 4).

**Tabela 4:** Internações e coeficientes padronizados de internação (por 100 mil habitantes), estratificados por Unidade Federativa, Brasil, 2010–2019.

Variável	Internações		Coef. de internação^a^	
	
	n	%	Coef.	IC95%
Internações totais	1.044.227	100	51,3	51,0–51,6
Região Norte				
Rondônia	8.229	0,8	47,5	44,3–50,9
Acre	1.128	0,1	14,1	11,6–17,0
Amazonas	13.011	1,2	33,8	32,0–35,7
Roraima	804	0,1	16	12,7–19,9
Pará	28.602	2,7	35,1	33,8–36,4
Amapá	759	0,1	10,1	7,9–12,6
Tocantins	3.468	0,3	23,1	20,7–25,6
Região Nordeste				
Maranhão	14.572	1,4	21,2	20,1–22,3
Piauí	5.185	0,5	16,1	14,7–17,6
Ceará	33.625	3,2	37,7	36,5–39,0
Rio Grande do Norte	15.973	1,5	46,9	44,7–49,3
Paraíba	21.962	2,1	56,2	53,8–58,5
Pernambuco	56.022	5,4	60,1	58,5–61,7
Alagoas	13.359	1,3	40,5	38,4–42,7
Sergipe	4.633	0,4	20,6	18,8–22,6
Bahia	40.506	3,9	27,3	26,5–28,1
Região Sudeste				
Minas Gerais	168.546	16,1	81,3	80,0–82,5
Espírito Santo	17.937	1,7	46,3	44,2–48,5
Rio de Janeiro	84.965	8,1	50,9	49,8–52,0
São Paulo	267.710	25,6	60,4	59,7–61,1
Região Sul				
Paraná	68.893	6,6	62	60,5–63,5
Santa Catarina	37.116	3,6	54,6	52,8–56,4
Rio Grande do Sul	91.231	8,7	81,5	79,8–83,2
Região Centro-Oeste				
Mato Grosso do Sul	5.377	0,5	20,5	18,9–22,4
Mato Grosso	16.506	1,6	50,5	48,1–53,0
Goiás	13.647	1,3	20,5	19,4–21,7
Distrito Federal	10.461	1	36,6	34,4–38,9

Coef.: coeficiente; IC95%: intervalo de confiança de 95%.

^a^ Coeficiente anual médio obtido pelo método direto de padronização, onde o numerador foi o número médio (média de 2010 a 2019) de internações aplicadas à população de referência (2014) e o denominador foi a população do respectivo estrato, neste ano de referência. As informações populacionais por unidade federativa foram obtidas do Censo Demográfico 2010 do IBGE ou das estimativas da Pesquisa Nacional por Amostra de Domicílios (PNAD) para os anos intercensitários.

Na análise de tendências da taxa de mortalidade (Tabela 5), observou-se uma tendência geral de aumento em todo o período estudado (2010–2019, AAPC 7,4; IC95% 6,1–8,7). Duas principais tendências foram encontradas no país: uma tendência de aumento acentuado no período de 2010 a 2016 (APC 9,1; IC95% 7,5–10,8), seguida de uma tendência também crescente, mas menos acentuada, no período de 2016 a 2019 (APC 4,0; IC95% 0,2–8,0). De acordo com os estratos da população, observou-se que o padrão temporal nos sexos masculino e feminino acompanhou o padrão nacional, com um período de crescimento acentuado nos anos 2010–2016 (APC próximos ao nacional de 9,1) e um período de crescimento menos acentuado nos anos 2016–2019 (APC de 4,0 igual ao nacional). Quanto às faixas etárias, os idosos apresentaram o APC mais acentuado no período de 2010–2016 (APC 8,4; IC95% 7,2–9,6), enquanto no período de 2010–2014 a faixa etária de cinco a nove anos apresentou uma significativa redução da mortalidade (APC -6,8; IC95% -9,4 a -4,2). Quanto às regiões, o Nordeste se destacou, apresentando o maior aumento da mortalidade entre todas as regiões, no período 2010–2016, com APC 17,8 (IC95% 14,5–21,2). Observa-se que, em nenhuma região, houve tendência significativa de declínio da mortalidade.

**Tabela 5:** Análise de regressão por *joinpoints* dos coeficientes padronizados de mortalidade por sepse, estratificados por sexo, faixa etária, raça e Região, Brasil, 2010–2019.

Variável	Tendência 1			Tendência 2			Período total	
		
	Período	APC	IC95%	Período	APC	IC95%	AAPC	IC95%
Brasil – Total	2010–2016	9,1^a^	7,5 a 10,8	2016–2019	4,0^a^	0,2 a 8,0	7,4^a^	6,1 a 8,7
Sexo								
Feminino	2010–2016	9,2^a^	7,3 a 11,2	2016–2019	4,0	-0,7 a 8,8	7,4^a^	5,9 a 9,0
Masculino	2010–2016	9,1^a^	7,8 a 10,4	2016–2019	4,0^a^	0,8 a 7,2	7,3^a^	6,3 a 8,4
Grupo etário (anos)								
≤ 4	2010–2015	-2,6^a^	-4,3 a -0,8	2015–2019	-0,1	-2,9 a 2,8	-1,5^a^	-2,7 a -0,3
5 a 9	2010–2014	-6,8^a^	-9,4 a -4,2	2014–2019	3,1^a^	1,1 a 5,2	-1,4^a^	-2,7 a -0,2
10 a 19	2010–2016	3,0^a^	2,2 a 3,7	2016–2019	-2,3	-5,1 a 0,6	1,2^a^	0,4 a 2,0
20 a 29	2010–2015	7,2^a^	4,1 a 10,4	2015–2019	-0,3	-4,1 a 3,7	3,8^a^	2,0 a 5,7
30 a 39	2010–2015	5,8^a^	2,7 a 9,0	2015–2019	-0,8	-4,5 a 3,2	2,8^a^	1,0 a 4,7
40 a 49	2010–2016	4,8^a^	4,1 a 5,4	2016–2019	0,3	-1,6 a 2,2	3,3^a^	2,7 a 3,8
50 a 59	2010–2016	5,0^a^	3,6 a 6,4	2016–2019	0,4	-3,7 a 4,7	3,4^a^	2,2 a 4,7
≥ 60	2010–2016	8,4^a^	7,2 a 9,6	2016–2019	1,9	-1,4 a 5,2	6,2^a^	5,1 a 7,2
Raça								
Branca	2010–2019	7,9^a^	7,1 a 8,8				7,9^a^	7,1 a 8,8
Parda	2010–2019	12,0^a^	10,1 a 14,0				12,0^a^	10,1 a 14,0
Preta	2010–2013	2,4	-16,9 a 26,3	2013–2019	12,8^a^	8,2 a 17,6	9,2^a^	3,1 a 15,7
Grande Região								
Norte	2010–2019	7,7^a^	5,8 a 9,7				7,7^a^	5,8 a 9,7
Nordeste	2010–2016	17,8^a^	14,5 a 21,2	2016–2019	1,1	-6,0 a 8,6	11,9^a^	9,4 a 14,6
Sudeste	2010–2015	8,1^a^	5,8 a 10,4	2015–2019	4,6^a^	2,0 a 7,3	6,5^a^	5,2 a 7,9
Sul	2010–2019	6,3^a^	5,8 a 6,9				6,3^a^	5,8 a 6,9
Centro-Oeste	2010–2016	8,2^a^	6,1 a 10,3	2016–2019	4,2	-1,2 a 9,9	6,9^a^	5,1 a 8,7

APC: mudança percentual média. AAPC: mudança percentual média anual. IC95%: intervalo de confiança de 95%.

^a^ Significativamente diferente de zero (p < 0,005).

## DISCUSSÃO

A sepse é um grave problema de saúde no Brasil e no mundo, sendo um desafio a ser enfrentado pelas políticas públicas. Estudos recentes demonstraram a tendência de aumento dessa síndrome no cenário nacional, evidenciando diversos fatores que contribuem para esse contexto. Dentre eles, destaca-se o aumento da população brasileira concomitantemente ao aumento da expectativa de vida, fato que expõe uma maior quantidade de pacientes com doenças crônicas e imunossuprimidos^14,15^.

Em relação à mortalidade, no mesmo período, foram notificados 462.971 óbitos pela doença, com coeficiente de mortalidade de 22,8 óbitos por 100 mil habitantes (IC95% 22,6–23,0). Diversos motivos podem estar associados à alta taxa de mortalidade por sepse no Brasil, destacando-se a possível negligência dada por profissionais de saúde aos quadros de sepse, o que dificulta e atrasa o tratamento da doença, ocasionando o aumento da mortalidade. Ademais, outro fator associado seria a ausência de unidades de cuidados intermediários nos hospitais brasileiros, o que levaria a uma permanência maior dos pacientes na UTI, levando a uma maior exposição e prevalência da sepse^3^.

Comparando-se a outros países, tanto desenvolvidos quanto subdesenvolvidos, as mortes por sepse no Brasil estão em uma tendência global da alta prevalência. Uma pesquisa feita na Espanha mostrou uma tendência de aumento de casos de sepse no país e uma prevalência de internações de 57 casos a cada 100 mil habitantes, número semelhante aos achados pelo presente estudo sobre a realidade brasileira (51,3 casos a cada 100 mil habitantes). Tal fato demonstra que, ainda que países desenvolvidos tenham melhores condições financeiras para arcar com gastos em saúde pública, a sepse se mostra uma problemática grave a ser enfrentada mundialmente^16^.

A pesquisa brasileira *Spread*, estudo multicêntrico conduzido pelo ILAS, avaliou a prevalência e a letalidade por sepse grave e choque séptico em 2015, quando esse aumento de mortalidade por sepse no Brasil foi mais nítido^3^. Ademais, analisando um estudo que verificou as taxas de óbitos por septicemia por regime nas regiões do Brasil, de 2011 a 2017, essas maiores taxas foram notadas em todas as regiões, exceto na Região Sul. A maior parte dos óbitos ocorrem no setor público, cerca de 60% das mortes, o que demonstra a necessidade de melhorias na saúde pública^17^. Além disso, a alta prevalência em alguns estados pode estar relacionada ao alto número de habitantes da região^18^, como São Paulo, o estado mais populoso do Brasil, onde ocorreram 25,6% dos casos de sepse.

Nesse estudo, 51 (23%) dos 227 hospitais analisados apresentavam baixa disponibilidade de recursos, o que elucida como o investimento reduzido nos meios necessários para o diagnóstico e tratamento da sepse interfere diretamente na mortalidade pela doença, fato exemplificado pelas regiões Sudeste, recebeu investimento de R$ 216.979.860,32, e Centro-Oeste, com R$ 15.798.077,40^18^.

Além disso, as regiões brasileiras com menores índices de internações foram as regiões Centro-Oeste (4,4%) e Norte (5,4%). Do mesmo modo, essas regiões representaram os menores coeficientes de mortalidade, totalizando 12,1 óbitos/100 mil habitantes da região Norte (IC95% 11,6–12,6) e 12,8 óbitos/100 mil habitantes da região Centro-Oeste (IC95% 12,2–13,4). Diante disso, um dos possíveis motivos para o menor número de internações e óbitos é a baixa densidade demográfica dessas regiões e o menor número de idosos (acima dos 60 anos) e, consequentemente, portadores de doenças crônicas que propiciam o desenvolvimento da sepse. Também é importante destacar a hipótese de subnotificação de internações e de óbitos por sepse, causando omissão de dados epidemiológicos importantes, visto que tais subnotificações podem estar associadas à falta de assistência médica, de recursos para diagnóstico de causa básica e laudos incorretos^19^.

Apesar de apresentar os menores índices de internações e de óbitos do país, a Região Centro-Oeste, segundo o estudo *Spread*, possui a taxa de mortalidade mais alta com 70%, enquanto a região Sudeste apresenta a mais baixa 51,2%. Nesse cenário, a relação existente entre as altas taxas de mortalidade por sepse no Brasil e os baixos níveis de investimentos em UTI foi demonstrada em um estudo epidemiológico publicado pelo *The Lancet,* o qual comprovou a associação entre baixos investimentos e alta taxa de mortalidade^3^.

Outrossim, cabe destacar os quantitativos da sepse nas regiões Nordeste e Sul. A Região Nordeste, segunda região com maior número de internações, totalizou 19,7% do contingente nacional, com coeficiente igual a 36,8 internações por 100.000 habitantes (IC95% 36,3–37,3), seguida pela região Sul, 18,9%, com coeficiente de internação igual a 67,8 a cada 100 mil habitantes (IC95% 66,8–68,7). Nesse contexto, a região Nordeste foi responsável pelo segundo maior número de mortes pela doença, 18,3% do total nacional, seguida pela região Sul, com 16,3% dos óbitos.

Dentre os estados da Região Nordeste, o maior percentual de internação foi identificado em Pernambuco (5,4%), perfazendo um coeficiente de mortalidade de 27,8 a cada 100 mil habitantes (IC95% 26,7–28,9). Fato que corrobora estudo epidemiológico com ano de referência de 2015, em que o estado também foi visto como o mais acometido, com 7.861 internações e 44,94% de taxa de mortalidade^18^. Além disso, pode-se observar uma notável diferença percentual entre os estados de Pernambuco e do Piauí quando se observa as taxas de internação, em que Pernambuco apresenta 5,4% das notificações, e o Piauí, 0,5%. Essa disparidade pode estar atrelada à diferença populacional entre os dois estados, tendo o estado de Pernambuco uma população aproximadamente duas vezes maior^20^.

No Brasil, não foi observada uma variação estatisticamente considerável no número de internações de homens e mulheres, contabilizando 52,4% dos casos do sexo masculino, contra 47,6% do sexo feminino. Os números referentes aos óbitos seguiram o mesmo padrão, o sexo masculino representou 51,4% e o feminino 48,6%. Considerando que o risco relativo de morte no sexo feminino foi de 1,05, não se pode afirmar que exista uma predominância de óbitos provenientes de qualquer um dos sexos. Uma revisão sistemática com cobertura de uma década da literatura, afirma que os estudos que apontam mortalidade relacionada a gênero e sepse são inconclusivos^21^. Ao analisar a literatura, alguns achados apontaram prevalência masculina, incluindo um estudo retrospectivo de abordagem quantitativa, realizado no Brasil, em que a prevalência do sexo masculino foi percebida e documentada em uma amostra com 347 pacientes^22^. Nesse sentido, entende-se que o presente estudo corrobora a acurácia estatística no cenário científico brasileiro.

O padrão temporal das tendências da taxa de mortalidade de ambos os sexos apresentou crescimento acentuado durante todo o período analisado, evidenciando concordância com o padrão nacional (2010–2019, AAPC 7,4; IC95% 6,1–8,7).

Esses dados divergem de um estudo da análise da incidência mundial por sepse entre 1990 e 2017, no qual constatou-se uma regressão no número de óbitos durante o período avaliado, evidenciando que no período pesquisado a incidência da sepse diminuiu 18,8% e a mortalidade por sepse diminuiu 29,7%. Nesse mesmo artigo, percebe-se uma grande variação das taxas de mortalidade por sepse entre diferentes países (América Latina e África apresentam os maiores índices) o que é explicado pela ineficácia dos sistemas de saúde dos locais com mais óbitos em prevenir, identificar e tratar a sepse, apesar da necessidade de mais estudos para melhor compreender essa disparidade^23^.

Além disso, em países com alta incidência de sepse, como o Brasil, há a suspeita de que muitos dos casos sejam devido à infecção nosocomial (pacientes que adquirem infecções nos hospitais), devido a procedimentos invasivos ou por conta de higienização inadequada das mãos de profissionais de saúde. Com isso, presume-se que o Brasil ainda se encontra com projeções negativas em relação aos parâmetros globais, sendo necessárias medidas para identificação, controle e prevenção de sepse.

Ao analisar a faixa etária dos indivíduos que evoluíram a óbito por sepse, o maior índice corresponde ao grupo acima de 60 anos de idade (70,8%), perfazendo um coeficiente de mortalidade de 114,1 óbitos por 100 mil habitantes e um risco relativo de 5,65 (IC95% 5,40–5,91), seguido do grupo de 50 a 59 anos (12%), com 24 óbitos por 100 mil habitantes. Em concordância, em um estudo feito com a análise de 848 pacientes admitidos na UTI com sepse grave e choque séptico, os pacientes idosos representaram 62,6% dos indivíduos internados e com o maior escore APACHE III (*Acute Physiology and Chronic Health Evaluation*), além de maior tempo de internação, o que é explicado pela maior presença de doenças crônicas, comorbidades, fragilidade e comprometimento funcional nesses indivíduos^24^.

Ademais, o grupo dos idosos apresentou uma tendência geral de aumento da mortalidade entre 2010 e 2019 (AAPC 6,2; IC95% 5,1–7,2). Nesse âmbito, uma revisão de literatura acerca da epidemiologia da sepse nos Estados Unidos atestou que mais da metade dos casos de sepse grave ocorre em indivíduos com mais de 65 anos, isso porque essa população é a mais acometida por doenças crônicas, aumentando o risco da mortalidade por sepse^25^.

Pode-se ainda afirmar que o envelhecimento, ao diminuir a produção de citocinas e causar alterações na imunidade adaptativa, deixa os indivíduos mais vulneráveis ao quadro séptico. Além disso, mulheres em idade pós-menopausa, devido à queda de estrogênio, apresentam alterações estruturais do aparelho geniturinário, ficando mais suscetíveis a desenvolver infecção do trato urinário, que, de acordo com o estudo *Spread*, representa uma das quatro principais causas de infecção que evolui para sepse, acompanhada da infecção pulmonar e intra-abdominal^26,3^. Fica evidente, portanto, a tendência de aumento da mortalidade de idosos, no cenário nacional, tendo em vista o envelhecimento da população e a exposição aos fatores de risco^14^.

No grupo etário menores de quatro anos, observa-se um número considerável de internações, 14,9% do total, ficando atrás somente dos maiores de 60 anos (53%), e um valor importante, mas menos expressivo, no número de óbitos, com 3,7% do total, ficando atrás dos grupos etários acima de 40 anos. Do mesmo modo, uma meta-análise, realizada em 2018, em que foi avaliado o impacto global da sepse pediátrica e neonatal entre 1979 e 2016, postulou que a mortalidade em crianças e neonatos variou entre 1% e 5%, em casos de sepse, e entre 9% e 20% em sepse severa^27^. Além disso, um seminário online envolvendo especialistas em infectologia pediátrica, em 2017, indicou que nos Estados Unidos a incidência da sepse neonatal é de um a quatro em mil nascidos vivos, além de relacionar o menor peso ao nascer com a maior probabilidade de desenvolver um quadro séptico (10,96 casos a cada mil nascidos vivos para recém-nascidos que pesavam entre 401 e 1.500g)^28^. Com isso, percebe-se a quantidade expressiva de internações por sepse neonatal e pediátrica no Brasil, apesar de a mortalidade encontrar-se dentro dos parâmetros mundiais, sendo necessária a atenção das instituições de saúde para esses parâmetros.

Ao analisar a variável racial, considerando as raças branca, preta e parda, em relação ao coeficiente de mortalidade, a maior taxa ocorreu na raça branca, seguido de pardos e pretos. Nesse mesmo cenário, uma pesquisa realizada em 60 municípios das cinco regiões brasileiras, em 2017, baseada no Sistema de Informação sobre Mortalidade, revelou incidência de 54,6% do total de óbitos por sepse em indivíduos de raça branca, seguido da raça parda (33,7%) e preta (8,2%)^9^.

Entretanto, uma revisão integrativa de literatura sobre o acesso da população negra a serviços de saúde, constatou que esse grupo racial sofre maiores restrições no acesso a serviços de saúde por diversos fatores como discriminação, violência, barreiras estruturais, fatores socioeconômicos, atuação dos profissionais, desrespeito à diversidade cultural, étnica e racial. Consequentemente, muitos dados sobre essa parcela da população não constam na base de dados, o que pode tornar a leitura e interpretação dos dados referentes a esse grupo parcialmente deturpadas^29^.

Ademais, segundo o estudo *Infection Rate and Acute Organ Dysfunction Risk as Explanations for Racial Differences in Severe Sepsis*, a mortalidade entre os pacientes pretos internados por infecção e sepse grave foi maior que em brancos, explicada por maior probabilidade de internação com infecção e maior risco de desenvolver disfunção aguda de órgãos^30^. Tal discrepância de dados, comparando os EUA e o Brasil, possivelmente justifica-se pela grande miscigenação da população brasileira, além do grande quantitativo de notificações sem informações no quesito raça/cor e do menor acesso de pretos ao sistema de saúde.

Sendo assim, é importante levar em consideração que o Brasil é um país extremamente heterogêneo, por isso os dados sobre internações por sepse tendem a variar de acordo com as características regionais, dado o número de habitantes, o valor de investimento proposto em cada unidade federativa e/ou as diferenças socioeconômicas de cada região. Ao realizar o trabalho, foram encontradas divergências em relação a outros estudos e às disparidades vistas em um país como o Brasil, o que pode ocorrer caso a base de dados não esteja com todas as informações atualizadas.

Por fim, neste estudo pretendeu-se ampliar o conhecimento sobre o perfil do paciente séptico e o desenvolvimento da doença em todo o país. Dessa forma, percebeu-se que mais estudos são necessários a fim de correlacionar esses achados e, assim, gerar maior contribuição e embasamento para políticas públicas e privadas de prevenção da sepse, assistência precoce e, consequentemente, redução da mortalidade, principalmente para os grupos mais acometidos pela doença, garantindo qualidade de vida para a população.
